# Supplementary material for: Enhanced Acetone Sensing Property of a Sacrificial Template Based on Cubic-Like MOF-5 Doped by Ni Nanoparticles
Source: Nanomaterials (Basel). 2020 Feb 22;10(2):386. doi: 10.3390/nano10020386 (PMC7075315; doi:10.3390/nano10020386)
Supplement: Supplementary file 1 [file nanomaterials-10-00386-s001.pdf]

# Supporting Information

# Enhanced Acetone Sensing Property of a Sacrificial Template Based on Cubic-Like MOF-5 Doped by Ni Nanoparticles

**Ning Zhang [1], Huijun Li [1,*], Zhouqing Xu [1,*], Rui Yuan [1], Yongkun Xu [1] and Yanyu Cui [1]**

[1] Department of Chemistry and Chemical Engineering, Henan Polytechnic University, Jiaozuo 454000, China

* Correspondence: lihuijunxgy@hpu.edu.cn (H.L.); zhqxu@hpu.edu.cn (Z.X.); Tel.: +0391-3986824 (H.L. & Z.X.)

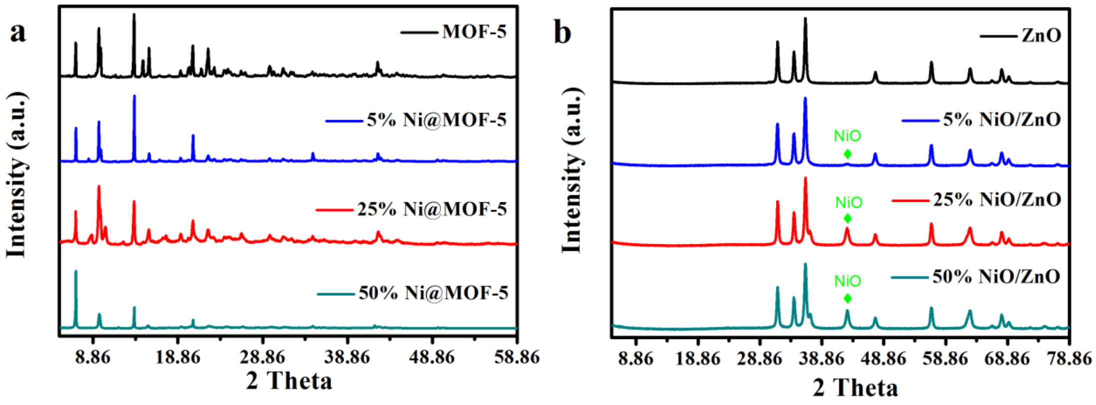


**Figure 1S.** The PXRD pattern of (a) MOF-5, 5% Ni@MOF-5, 25% Ni@MOF-5 and 50% Ni@MOF-5, (b) ZnO, 5% NiO/ZnO, 25% NiO/ZnO and 50% NiO/ZnO.

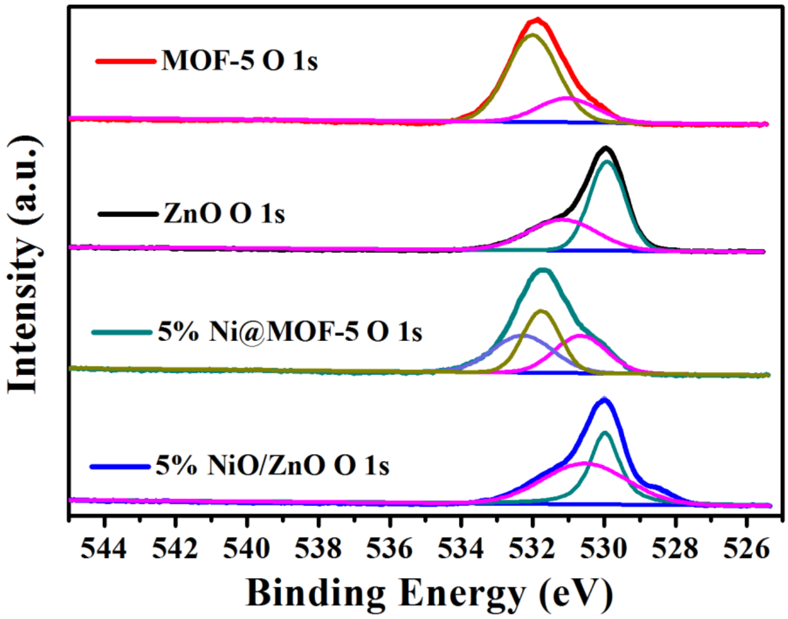


**Figure 2S.** O 1s spectrum of materials.

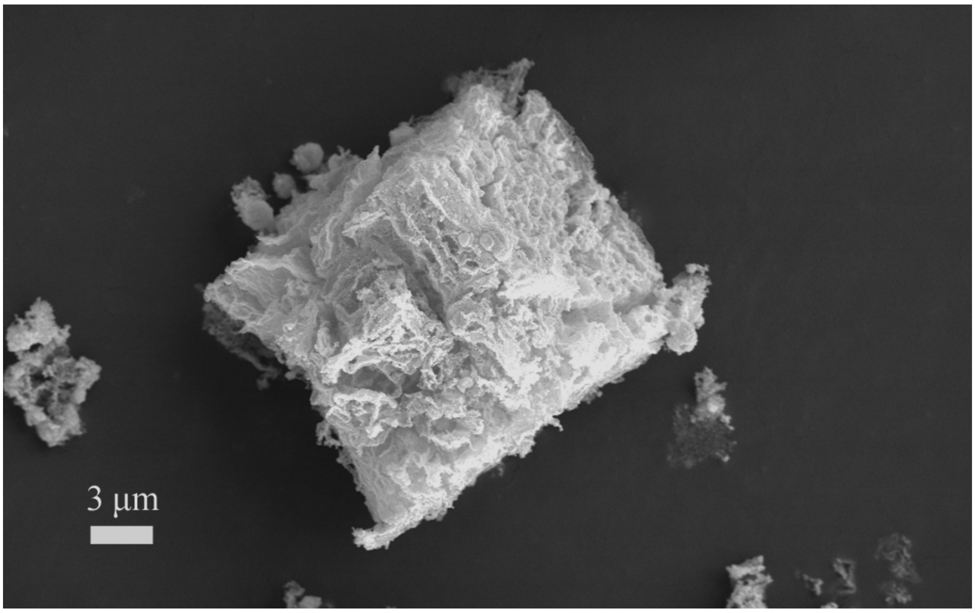


**Figure 3S.** SEM images of 25% NiO/ZnO.

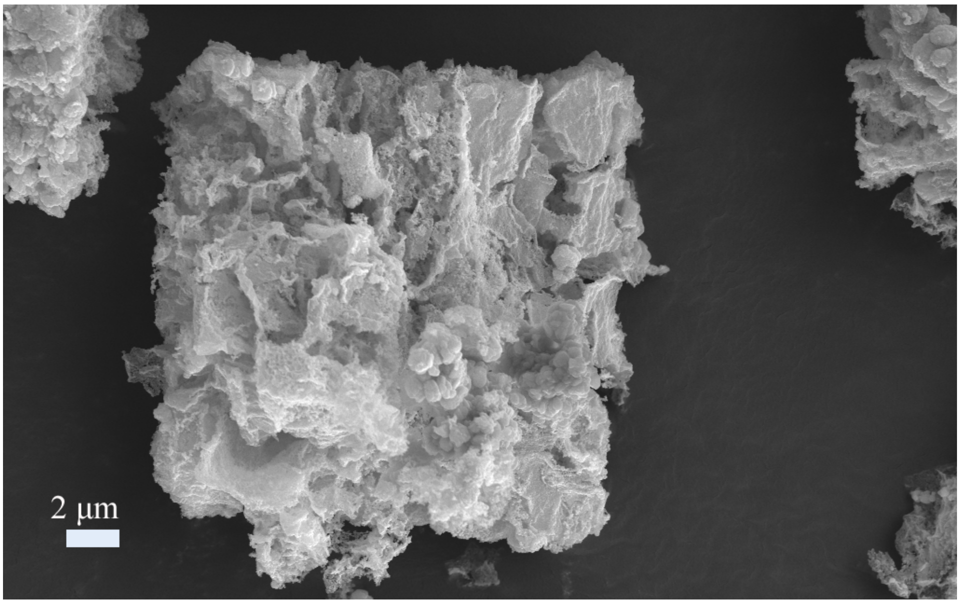


**Figure 4S.** SEM images of 50% NiO/ZnO.

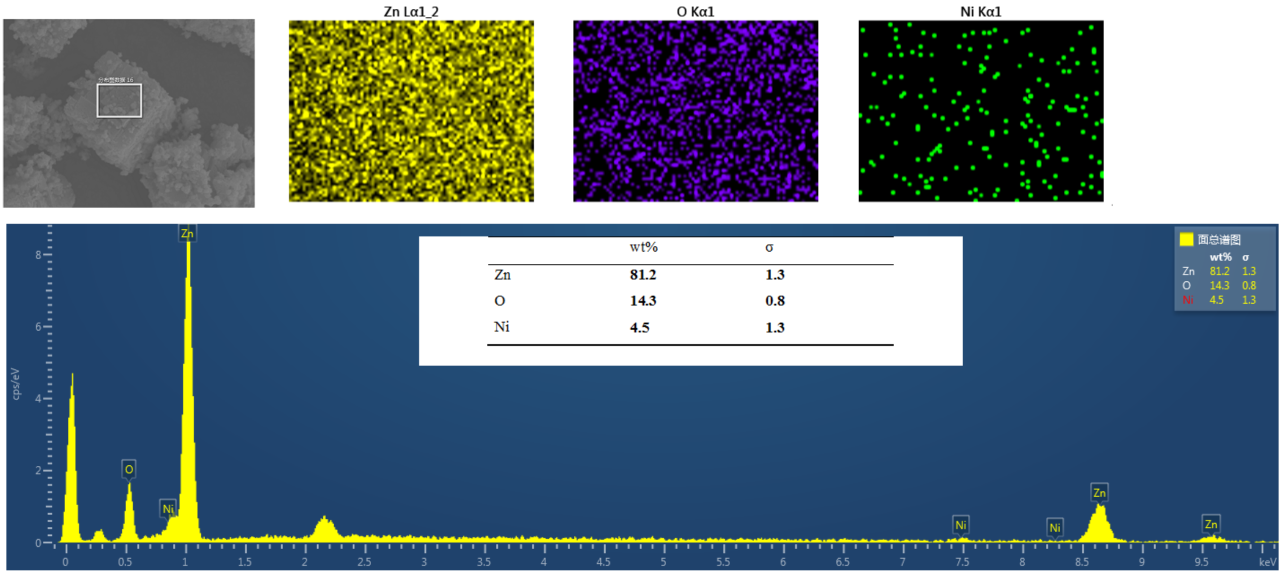


**Figure 5S.** EDS spectrum of 5% NiO/ZnO microstructure.

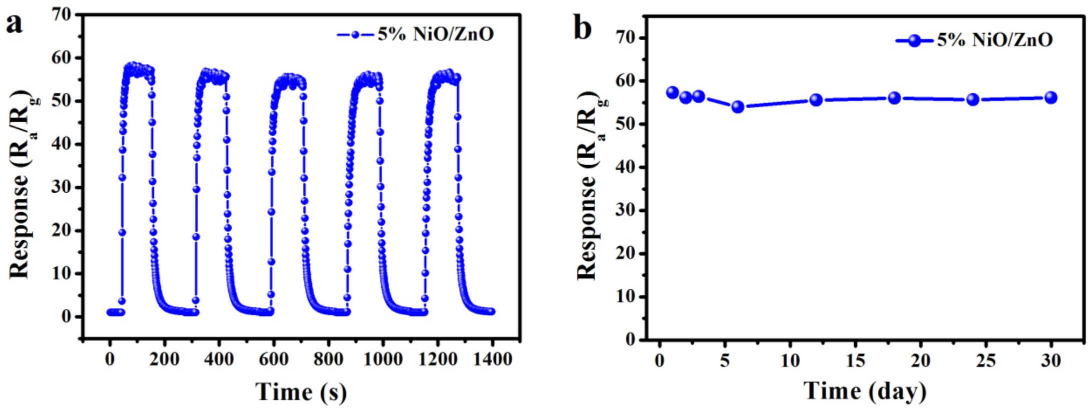


**Figure 6S.** (a) Repeatability measurement of the 5% NiO/ZnO to 200 ppm acetone at 340°C, (b) The long-term stability test of 5% NiO/ZnO to acetone (200 ppm).
